# Coronary Artery Embolism and Myocardial Infarction, case report

**DOI:** 10.47487/apcyccv.v5i1.347

**Published:** 2024-03-19

**Authors:** Alejandro Narváez Orozco, Alberto Navarro Navajas, Carolina Cardona Buitrago, Juan M. Senior-Sánchez, Juan Camilo Ortiz Uribe, Juan Andrés Delgado Restrepo

**Affiliations:** 1 Universidad de Antioquia, Hospital Universitario San Vicente Fundación, Antioquia, Colombia. Universidad de Antioquia Universidad de Antioquia Hospital Universitario San Vicente Fundación Antioquia Colombia; 2 Sección de Cardiología, Departamento de Medicina Interna, Grupo para el Estudio de las Enfermedades Cardiovasculares, Universidad de Antioquia. Antioquia, Colombia. Universidad de Antioquia Sección de Cardiología, Departamento de Medicina Interna, Grupo para el Estudio de las Enfermedades Cardiovasculares Universidad de Antioquia Antioquia Colombia; 3 Servicio de Hemodinamia, Unidad Funcional Integrada Cardiopulmonar y Vascular Periférica, Hospital Universitario San Vicente Fundación, Antioquia, Colombia. Servicio de Hemodinamia, Unidad Funcional Integrada Cardiopulmonar y Vascular Periférica Hospital Universitario San Vicente Fundación Antioquia Colombia

**Keywords:** Myocardial Infarction, Acute Coronary Syndrome, Embolism, Coronary Artery, Atrial Fibrillation

## Abstract

Coronary embolism (CE) is a rare cause of non-atherosclerotic acute coronary syndrome (ACS). The clinical presentation is similar to ACS, and the diagnosis is supported by Shibata criteria. Atrial fibrillation is the main reported etiology in CE cases. Management includes percutaneous intervention with thromboaspiration and anticoagulation. The following case is a description of a patient with acute chest pain and recently diagnosed atrial fibrillation (AF) with a rapid ventricular response, is described. A thrombotic lesion in the distal right coronary artery (RCA) of embolic origin, was documented. Successful mechanical thromboaspiration was performed; intravascular ultrasound (IVUS) showed no thrombus, dissection, or atherosclerotic plaque. CE is an underdiagnosed cause of ACS; diagnosis relies on Shibata criteria, and patients experience worse outcomes in follow-up.

## Introduction

The coronary embolism (CE) is an uncommon cause of myocardial infarction (MI). A prevalence of 3% of cases of acute coronary syndrome (ACS) has been reported, although post-mortem studies show a prevalence of up to 13% ^1^^,^^2^. Atrial fibrillation (AF) is the most frequently associated pathology with the occurrence of CE. The anterior descending artery (ADA) is the vessel most commonly affected, and the presentation is similar to that of atherosclerotic ACS. It predominantly affects women and has similar traditional risk factors. In most cases, it is diagnosed using coronary angiography (CA) and Shibata criteria ^3^^,^^4^. Despite the recognition of the condition, it is considered an underdiagnosed cause of ACS, and there is uncertainty regarding the standard short- and long-term management of these patients ^1^^,^^3^^,^^4^. This article aims to present the pathophysiology, diagnosis, management and prognosis through the presentation of a case report.

## Case report

A 76-year-old woman with a history of hypertension was admitted for chest pain with cardiac characteristics of 4 hours of evolution, associated with palpitations. Physical examination showed irregular pulse, heart rate of 120 beats per minute; other vital signs and physical examination showed no abnormalities. The initial electrocardiogram showed atrial fibrillation with rapid ventricular response and 4 mm ST segment elevation in inferior wall leads. She was managed with aspirin, clopidogrel, and enoxaparin.

**Figura 1:**
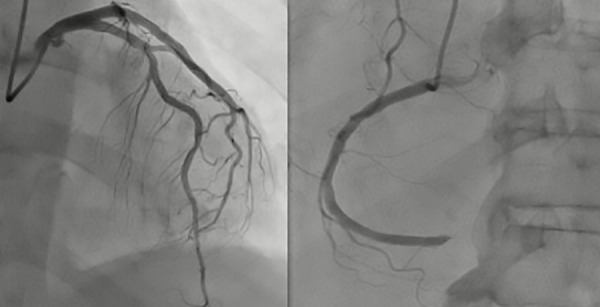
Initial Coronary Angiography. A) Left main coronary artery, left anterior descending artery, and left circumflex artery without significant stenotic lesions. B) Right coronary artery occluded in the distal segment.

**Figura 2:**
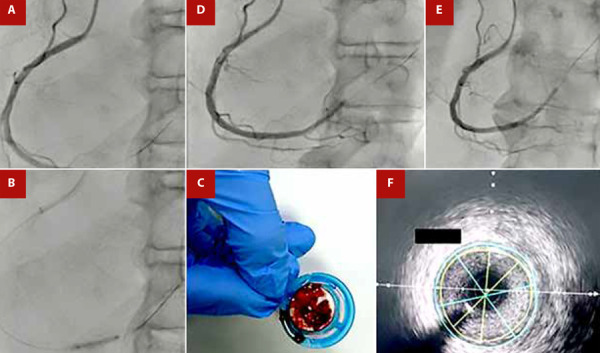
A) Initial Coronary Angiography showing thrombotic occlusion in the right coronary artery. B) Balloon angioplasty with a semi-compliant balloon of 2.0 x 15 mm up to 14 atm. C) Mechanical thrombus aspiration obtaining abundant thrombus material. D) and E) Post-mechanical thrombus aspiration with improvement of distal flow. F) Intravascular ultrasound (IVUS) in the right coronary artery performed at 48 hours showing no evidence of atherosclerotic lesion, thrombus, or dissection.

In the CA, the distal segment of the right coronary artery (RCA) was found to be occluded by thrombus, while the rest of the coronary arteries did not show significant lesions (Figure 1). The patient received 6000 units of unfractionated heparin intravenously and a bolus of 28 mL of intracoronary tirofiban, followed by an infusion of 7 mL/hour for 48 hours. Subsequently, angioplasty was performed using a 2.0 x 1.5 mm semi-compliant balloon up to 14 atm with no improvement in flow; then manual mechanical thrombus aspiration was performed with an EXPORT® catheter, obtaining abundant thrombotic material and improving distal flow. A control CA at 48 hours with intravascular ultrasound (IVUS) showed a RCA without evidence of atherosclerotic lesion, residual thrombus, or dissection, with TIMI 3 distal flow (Figure 2). Transthoracic echocardiogram revealed an ejection fraction of 44% and hypokinesia in the inferior wall. The patient’s condition improved, spontaneously converted to sinus rhythm, and was discharged with carvedilol 6.125 mg every 12 hours and apixaban 5 mg every 12 hours, while antiplatelet medication was discontinued. At 6-month follow-up, the patient remained in sinus rhythm and reported no symptoms.

## Discussion

CE is a rare cause of non-atherosclerotic ACS, while AF is the most common cause of CE ^1^. The prevalence ranges from 4 to 13% according to angiographic studies or autopsy-based studies, respectively ^2^^,^^3^. Physiologically, coronary arteries are anatomically protected against embolic events compared to the rest of the circulatory system. Most emboli generated during AF go to the central nervous system rather than the coronary arteries due to the hemodynamic characteristics of the system. Firstly, the diameter of the coronary vessels and the difference in vascular resistance between the aorta and the coronary arteries favor flow along the aorta and reduce the likelihood of directing toward the coronary circulation. Secondly, the coronary arteries originate from the aortic root, and their orifices are partially shielded by the aortic valves and the sinus of Valsalva. Finally, coronary flow mainly occurs during diastole (due to pressure differences between the aorta and the ventricles), especially in the left system, while emboli typically mobilize during systole ^5^.

The clinical presentation of CE is similar to that of atherosclerotic ACS, and diagnosis relies on clinical and imaging criteria. In 2015, Shibata *et al*., in a cohort of 1776 patients with ACS including 52 cases of CE, proposed major and minor criteria (Table 1). The presence of two or more major criteria, one major and more than two minor criteria, or all three minor criteria make a definitive diagnosis of CE, while a probable diagnosis is made if one major and one minor criterion or two minor criteria are present ^4^. In this cohort, AF accounted for 73% of the causes, followed by dilated cardiomyopathy (25%), presence of valvular prosthesis (7.7%); while distant embolism and septic embolism occurred in 3.8% of cases, similar to what was reported by Popovic, where AF was the most common cause followed by dilated cardiomyopathy. Other reported causes included cardiac tumors, antiphospholipid syndrome, and malignancy, in addition to the etiologies reported by Shibata ^6^. In both cohorts, only 62% of patients were diagnosed with definitive CE, highlighting the difficulty in confirming the diagnosis. Additionally, it should be noted that patients with coronary ectasia, history of coronary revascularization, >25% stenosis in non-culprit arteries, and plaque disruption evidenced on intravascular imaging were excluded from the studies ^4^^,^^6^^,^^7^. The described case met two major criteria and two minor criteria, thus CE was considered a definitive diagnosis.

**Table 1:** Criteria for the definitive diagnosis of coronary embolism

Major Criteria	Minor Criteria
a) Angiographic evidence of coronary embolism and thrombosis without atherosclerotic component b) Concomitant coronary embolization: multiple vessels in the same coronary territory or multiple vessels of the coronary tree c) Concomitant systemic embolism without evidence of intracavitary thrombus attributed to acute myocardial infarction	a) Stenosis <25% on coronary angiography, except in the culprit lesion b) Evidence of an embolic source by multimodal imaging c) Presence of embolic risk factors such as atrial fibrillation, dilated cardiomyopathy, rheumatic valve disease, presence of valve prosthesis, patent foramen ovale, atrial septal defects, infective endocarditis, or hypercoagulable state

Two or more major criteria, one major and more than two minor criteria, or all three minor criteria make a definitive diagnosis of coronary embolism, and a probable diagnosis would be made if one major and one minor criterion or two minor criteria are present..

Soliman *et al.* proposed a bidirectional relationship between AF and ACS. Inflammation, prothrombotic state, and rapid ventricular response may increase the risk of myocardial infarction (MI) ^7^; however, most AF-related extracranial embolisms will not affect the coronary arteries due to their particular anatomy and physiology. In a study involving 37,973 patients with AF, 221 cases of systemic embolism were reported, of which 11.5% corresponded to extracranial embolism. Lower limb vasculature was affected in 58% of cases, followed by visceral-mesenteric in 31% and upper limbs in 10%, with no cases of embolism to the coronary arteries found ^8^. These findings contrast with those of Shibata and Popovic, who conducted a systematic search for extracranial systemic embolism and were able to demonstrate it in 23% of cases, with the most common sites being the lower limbs and the kidney ^4^^,^^6^.

The coexistence of cerebral and coronary embolism is rare, reported in 0.25% of patients with ACS or acute stroke ^9^; however, it is believed that the prevalence is much higher. When systematically searched, the prevalence may be as high as 16-25%. Some identified factors that limit diagnosis include: the disappearance of coronary lesions due to thrombolysis, difficulty in interpreting electrocardiographic changes during stroke, and the presence of non-visualized distal embolisms on arteriography ^10^. In the absence of neurological symptoms and systemic embolism, a systematic search was not considered; however, as previously stated, its determination can increase diagnostic certainty.

Histopathological studies of the thrombus do not provide information about the pathophysiological mechanism of infarction, although some authors suggest that the study could help in the diagnosis of septic and malignancy-related embolism ^6^^,^^11^. From a clinical perspective, embolic origin MI should be considered in patients with compatible electrocardiographic changes ^12^, risk factors for embolism, presence of systemic embolism, and demonstration of an embolic source. Additionally, the importance of intravascular imaging in ruling out atherosclerotic causes is highlighted; in studies of patients with MI without atherosclerotic lesions on CA, IVUS was able to diagnose plaque disruption in 38% of patients ^10^^,^^11^.

Regarding interventional treatment in ACS of embolic etiology, thrombus aspiration, balloon angioplasty in certain scenarios, and the use of glycoprotein IIb/IIIa inhibitors (GPIIbIIIa) are the standard management, without the need for stent implantation. Control CA with intracoronary imaging (IVUS or optical coherence tomography) is recommended to rule out other underlying causes such as dissection, rupture, or erosion of atherosclerotic plaque ^13^, which is essential for long-term antithrombotic therapy with anticoagulant alone, antiplatelets, or both. In this patient, causes other than coronary embolism were ruled out with IVUS, and long-term anticoagulation therapy was offered.

In follow-up, these patients have a higher risk of cardiovascular and cerebrovascular events, leading to a worse prognosis with a higher incidence of all-cause death and cardiac mortality ^4^. In conclusion, CE due to AF is an uncommon cause of ACS of non-atherosclerotic etiology, owing to the protection of the coronary tree against embolic events; however, it is an underdiagnosed entity. Clinical suspicion begins with the presence of risk factors for embolism or evidence of an embolic source, and diagnosis is supported by Shibata criteria. Thrombus aspiration, anticoagulation, and glycoprotein IIb/IIIa inhibitors are the mainstays of management. These patients have worse outcomes in follow-up compared to the general population.
